# Genetic and sex-specific regulation of mitochondrial function in gonadal and inguinal adipose tissue

**DOI:** 10.1016/j.molmet.2025.102227

**Published:** 2025-08-05

**Authors:** Dorota Kaminska, Calvin Pan, Laurent Vergnes, Ashlyn Ro, Gurugowtham Ulaganathan, Aldons J. Lusis

**Affiliations:** 1 Department of Medicine, Division of Cardiology, David Geffen School of Medicine at UCLA, Los Angeles, CA, USA; 2 Department of Human Genetics, David Geffen School of Medicine at UCLA, Los Angeles, CA, USA; 3 Department of Microbiology, Immunology and Molecular Genetics, David Geffen School of Medicine at UCLA, Los Angeles, CA, USA

**Keywords:** iWAT, gWAT, Mitochondria, Metabolism, Sex

## Abstract

**Objective:**

Sex differences in adipose tissue impact metabolic health, but the underlying molecular mechanisms remain unclear. We previously identified a female-specific chr17 *trans*-eQTL hotspot regulating mitochondrial gene expression in gonadal white adipose tissue (gWAT). Here, we tested whether iWAT contributes comparably to sex differences in mitochondrial function and futile cycling.

**Methods:**

We analyzed iWAT and gWAT from male and female mice across 58 genetically diverse Hybrid Mouse Diversity Panel (HMDP) strains fed a high-fat, high-sucrose diet. We assessed mitochondrial DNA (mtDNA), oxidative phosphorylation (OXPHOS) and futile cycle gene expression, performed genetic mapping, and measured respiration.

**Results:**

In gWAT, females showed higher mtDNA, OXPHOS expression, and a female-specific chr17 trans-eQTL, correlating with metabolic traits. In contrast, iWAT lacked this hotspot and showed higher mtDNA, OXPHOS expression, and respiration in males. Lipid cycling genes (*Lipe*, *Mgll*, *Pnpla2*) were elevated in male iWAT, while *Mpc1*, *Mpc2*, and *Pck1* were enriched in female gWAT. *Ucp1* was higher in female gWAT but not sex-biased in iWAT. *Alpl* (TNAP), key creatine cycling gene, was upregulated in females in both depots, particularly in iWAT.

**Conclusions:**

Female gWAT shows genetically driven mitochondrial regulation linked to metabolic protection, whereas male iWAT has higher mitochondrial content, OXPHOS expression, and respiration. Elevated lipolytic enzymes in male iWAT suggest greater FFA release, while higher pyruvate import and glyceroneogenesis genes in female gWAT favor FFA recycling. *Alpl* upregulation in females indicates sex-biased UCP1-independent thermogenesis. These depot- and sex-specific signatures reflect distinct metabolic strategies and highlight the need to consider both in adipose research.

## Highlights

• Opposing patterns in gWAT and iWAT, with higher mtDNA, OXPHOS, and respiration in female gWAT and male iWAT.
• iWAT lacks the female chr17 *Ndufv2* trans-eQTL hotspot found in gWAT.
• Male iWAT is enriched for *Lipe*, *Mgll*, *Pnpla2*, suggesting greater lipid cycling.
• Female iWAT is enriched for *Alpl*, supporting UCP1-independent creatine thermogenesis.

## 1 Introduction

Adipose tissue is a heterogeneous and dynamic metabolic organ essential for energy storage, thermoregulation, and endocrine signaling. White adipose tissue (WAT), the primary energy storage site, is anatomically and functionally heterogeneous, with distinct depots contributing differently to metabolic health. In humans, subcutaneous fat is primarily localized in the abdominal, gluteal, and femoral regions, whereas in mice, the inguinal white adipose tissue (iWAT) is the principal subcutaneous fat pad and corresponds anatomically to the large gluteofemoral subcutaneous depot in humans [1]. Conversely, omental fat is the main visceral depot in humans but it is negligible in mice. In mice gonadal white adipose tissue (gWAT) serves the main visceral depot in mice, though it lacks a direct human counterpart [1,2].

Obesity is closely linked to a range of cardiometabolic disorders, including insulin resistance, type 2 diabetes, and metabolic dysfunction-associated steatotic liver disease (MASLD), formerly known as non-alcoholic fatty liver disease (NAFLD). Notably, the incidence and severity of these disorders differ markedly between sexes, with females generally exhibiting a more favorable metabolic profile than males. Sex differences in adiposity and fat distribution underlie variations in cardiometabolic risk, with visceral fat accumulation (more common in males) being more strongly associated with metabolic dysfunction, while subcutaneous adipose tissue, particularly in gluteofemoral regions (seen more in females), is considered to be protective [3].

Our prior work demonstrated that female gWAT exhibits higher mitochondrial content, elevated expression of oxidative phosphorylation (OXPHOS) genes, and enhanced oxidative capacity-all under genetic regulation by a female-specific *trans*-eQTL hotspot on chromosome 17 [4]. *Trans*-eQTL hotspots are genomic loci where genetic variants influence the expression of multiple genes located on other chromosomes, often pointing to central regulators. The chromosome 17 hotspot identified in gWAT affected a broad network of mitochondrial genes, suggesting coordination by a key upstream factor with sex-specific effects. *Ndufv2*, a core subunit of mitochondrial complex I, was identified as the causal effector gene at this locus [4]. These findings suggest that sex-specific genetic regulation of mitochondrial function in female adipose tissue may contribute to a more favorable metabolic profile. However, whether similar regulatory mechanisms exist in other depots remains unclear.

Mitochondrial gene expression exhibits marked tissue as well as sex specificity, as shown across various metabolically active tissues in recent animal studies [[5], [6], [7], [8], [9], [10], [11]]. These findings underscore the importance of examining depot-specific and sex-specific regulation in adipose tissue, which plays a key role in systemic energy metabolism. iWAT, in particular, is more prone to beiging than gWAT in response to cold exposure or exercise, suggesting it may have unique thermogenic and metabolic potential [12,13]. However, it remains unknown whether the sex-specific mitochondrial regulation previously observed in gWAT also applies to iWAT, and how the depot-specific differences in mitochondrial function may contribute to sex differences in systemic metabolic traits.

Given the established browning potential of iWAT and its proposed metabolic benefits [14,15] we hypothesized that it may also contribute to female metabolic advantage. Using the same panel of Hybrid Mouse Diversity Panel (HMDP) strains, we aimed to systematically compare mitochondrial function, gene expression, and genetic regulation in gWAT and iWAT, and to assess their potential contributions to sex-specific differences in metabolic traits.

## 2 Methods

### 2.1 Mouse cohort and diet

All mice used in this study were obtained from The Jackson Laboratory and subsequently bred in-house at UCLA. They were housed in an Institutional Animal Care and Use Committee (IACUC)-approved vivarium under controlled environmental conditions, including a 14-hour light/10-hour dark cycle (lights on from 06:00 to 20:00), temperature of 25 °C, and 30–70% relative humidity, with daily health monitoring to ensure animal welfare. All animal procedures were conducted in accordance with protocols approved by IACUC at UCLA (protocol #92-169).

The Hybrid Mouse Diversity Panel (HMDP) study design has been described in detail previously [16,17]. For this study, we analyzed 58 inbred HMDP strains for which matched male and female mice were available, each with paired gonadal (gWAT) and inguinal (iWAT) adipose tissue samples. Mice were maintained on a standard chow diet (Ralston Purina Company) until 8 weeks of age, then switched to a high-fat, high-sucrose (HF/HS) diet (Research Diets D12266B; 16.8% protein, 51.4% carbohydrates, 31.8% fat) for 8 weeks prior to tissue collection. On the day of tissue collection, mice were euthanized following a 4-hour fast, in accordance with approved institutional protocols.

All analyses were conducted using matched male and female samples from 58 HMDP strains (i.e., 58 males and 58 females) for both gonadal and inguinal adipose tissues. For eQTL mapping, a larger set of available strains was used to maximize statistical power: 103 female and 112 male strains for gWAT, and 67 female and 74 male strains for iWAT.

### 2.2 Mitochondrial DNA and gene expression analyses

Mitochondrial DNA (mtDNA) copy number was quantified by qPCR using a Roche LightCycler 480 with primers targeting the mitochondrial *D-loop* region (Forward: AATCTACCATCCTCCGTGAAACC; Reverse: TCAGTTTAGCTACCCCCAAGTTTAA) and normalized to the nuclear gene *Tert* (Forward: CTAGCTCATGTGTCAAGACCCTCTT; Reverse: GCCAGCACGTTTCTCTCGTT) [4]. gWAT expression was analyzed using Affymetrix HT_MG430A microarray [4], whereas the iWAT RNA-seq libraries were 100 bp paired-end, stranded, and sequenced to an average depth of 43 million reads per sample (1-4 samples per sex per strain). Transcript abundance was quantified using kallisto against the Ensembl release 97 transcriptome based on the Mus musculus GRCm38/mm10 reference genome. Kallisto output was imported into R using tximport to generate gene-level count estimates for downstream analysis. Differential gene expression analysis was performed using limma with the model ∼ Strain + Sex to identify main effects of sex across genetically diverse strains; interaction terms were not included, as the focus was on consistent sex differences rather than strain-specific interactions. Batch effects were evaluated and found to be negligible. Expression of mitochondrial genes was evaluated across MitoCarta3.0 mitochondrial transcripts.

### 2.3 Genetic mapping

Genotypic data for the mouse strains were generated using the Mouse Diversity Array as previously described [18]. Following quality control procedures and exclusion of variants with missing genotype information, approximately 200,000 high-quality single nucleotide polymorphisms (SNPs) were retained for analysis. The expression quantitative trait loci (eQTL) mapping was performed using FaST-LMM, incorporating a kinship matrix to control for population structure and relatedness. A leave-one-chromosome-out strategy was used to reduce proximal contamination. The genome-wide significance threshold was defined using empirical permutations, following the approach outlined in Kang et al. [19].

To increase statistical power for detecting genetic regulators of mitochondrial function, eQTL mapping of mitochondrial-related transcripts was performed in both gWAT and iWAT across HMDP strains. The analysis included 103 female and 112 male strains for gWAT, and 67 female and 74 male strains for iWAT. *Cis*-eQTLs were defined as SNPs within 1 Mb of the target gene; *trans*-eQTLs were mapped genome-wide. We focused on identifying *trans*-eQTL hotspots and tested for sex-specific effects.

### 2.4 Respirometry from frozen tissue

Mitochondrial respiration in frozen samples was performed on frozen adipose biopsies from SWR/J mice (*n* = 3 per sex per depot; 12 samples total) [20]. Briefly, the oxygen consumption rate was measured for complexes I (1 mM NADH), II (5 mM succinate), and IV (0.5 mM TMPD) in presence of 10 μg/ml cytochrome c in MAS (with 0.2% BSA) in a Seahorse Bioscience XF96 instrument.

### 2.5 Phenotype associations

Metabolic traits, including body weight, gWAT and iWAT mass, insulin, and HOMA-IR, were collected as previously described [16]. To assess associations between mitochondrial copy number and metabolic traits, we used Spearman rank correlation, a non-parametric method that does not assume a linear relationship or normal distribution and is less sensitive to outliers than parametric alternatives such as Pearson correlation.

## 3 Results

### 3.1 Male mice exhibit a more adverse metabolic profile despite comparable adiposity and fat distribution

**Figure 1:**
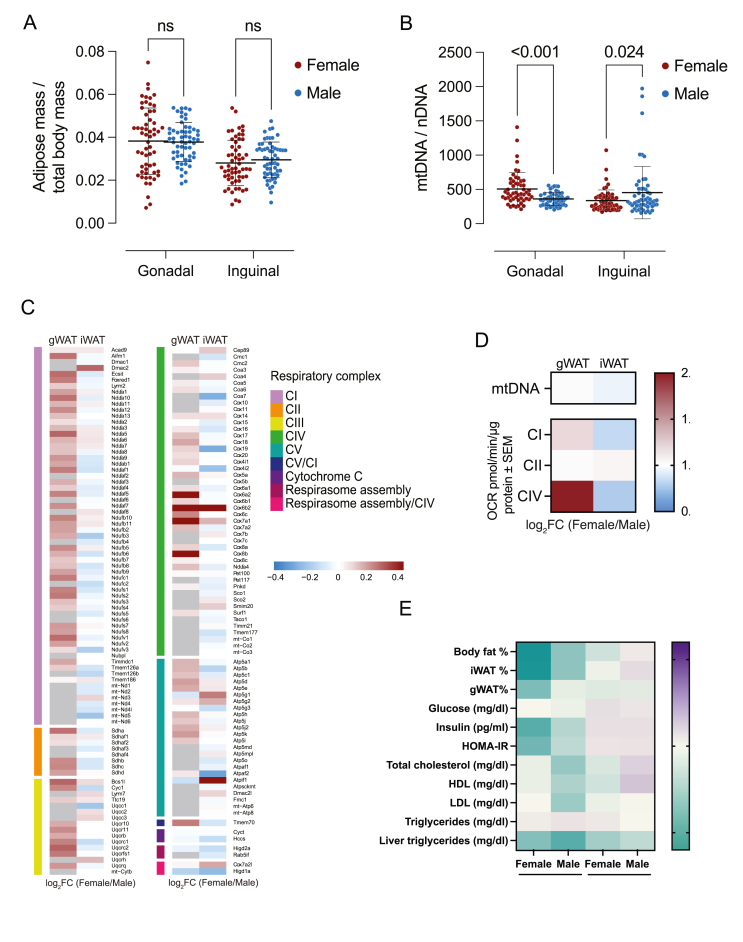
**Sex- and depot-specific differences in adiposity, mitochondrial content, and function across 58 HMDP strains. (A)** Relative adiposity of gWAT and iWAT depots (adipose mass/total body mass) in matched female and male mice. Paired Wilcoxon signed-rank tests were used. **(B)** Mitochondrial DNA (mtDNA) copy number in iWAT and gWAT, stratified by sex. Paired Wilcoxon signed-rank tests were used. **(C)** Heatmap showing sex differences in the expression of genes in the oxidative phosphorylation (OXPHOS) pathway. Differential expression was analyzed using limma with the model ∼ Strain + Sex to account for paired comparisons across matched strains. Values represent log_2_ fold change (female vs. male) in iWAT and gWAT. Red indicates higher expression in females; blue indicates higher expression in males; grey denotes genes not measured (gWAT) or not detected (iWAT). **(D)** Heatmap showing sex differences in mitochondrial respiration between gWAT and iWAT in SWR/J mice. Values represent log_2_ fold change (female vs. male) in mitochondrial respiration. Red indicates higher respiration in females; blue indicates higher respiration in males. Data are based on *n* = 3 males and *n* = 3 females per depot. **(E)** Correlation heatmap of mtDNA levels in gWAT and iWAT with metabolic traits. Traits and mtDNA values were log-transformed as needed to achieve normal distribution. Spearman’s correlation coefficients (rho) are shown separately for females and males. ∗*p* < 0.05, ∗∗*p* < 0.01, ∗∗∗*p* < 0.001. (For interpretation of the references to colour in this figure legend, the reader is referred to the Web version of this article.)

We first compared the overall metabolic status of male and female mice across 58 matched HMDP strains. While body fat percentage did not differ significantly between sexes (*p = 0.162*), males displayed a markedly more adverse metabolic profile. Fasting glucose was approximately 14% higher in males (*p < 0.001*), while insulin and HOMA-IR levels were elevated by over 140% and 170%, respectively (*p < 0.001 for both*), consistent with greater insulin resistance. Circulating lipids were also significantly elevated in males, with total cholesterol, HDL, and LDL increased by ∼48%, ∼54%, and ∼39%, respectively (*p < 0.001 for all*). Triglyceride levels were 24% higher in males (*p = 0.003*). Liver triglyceride content was nearly double in males compared to females (*p < 0.001*). In contrast, free fatty acid levels were comparable between sexes (*p* = 0.229). Median values and interquartile ranges for all traits are reported in Table 1. Moreover, we observed no clear sex differences in the relative distribution of iWAT (subcutaneous) or gWAT (visceral) fat depots (*gWAT: p = 0.160; iWAT: p = 0.933*) (Figure 1A), suggesting that total or depot-specific fat mass alone does not explain the observed sex-specific metabolic differences.

**Table 1:** Metabolic phenotypes in female and male mice from 58 matched inbred strains of the HMDP fed a high-fat, high-sucrose (HF/HS) diet.

Trait	Female	Male	*p*-value
Body fat (%)	30.2 [21.0, 36.6]	31.3 [27.1, 35.9]	0.162
Glucose (mg/dl)	198.7 [176.3, 219.3]	227.1 [191.9, 301.9]	<0.001
Insulin (pg/ml)	1339.8 [922.5, 2354.5]	3308.8 [2044.2, 5715.1]	<0.001
HOMA-IR	16.3 [8.9, 25.7]	45.1 [27.6, 89.8]	<0.001
Total cholesterol (mg/dl)	135.5 [114.4, 171.1]	200.1 [173.5, 224.5]	<0.001
HDL (mg/dl)	103.0 [89.8, 135.9]	158.6 [135.4, 179.8]	<0.001
LDL (mg/dl)	29.6 [24.8, 36.3]	41.2 [35.6, 47.5]	<0.001
Triglycerides (mg/dl)	38.2 [27.7, 50.8]	47.5 [38.4, 69.1]	0.003
FFA (mg/dl)	37.5 [33.3, 42.7]	37.3 [32.1, 41.2]	0.229
Liver triglycerides (mg/dl)	36.9 [26.7, 56.2]	73.4 [41.0, 109.1]	<0.001

Values are presented as median and interquartile range; *p* values reflect paired Wilcoxon signed-rank tests.

### 3.2 iWAT shows higher mitochondrial content in males, while females have higher mitochondrial content in gWAT

Building on our previous findings that females exhibit higher mitochondrial DNA (mtDNA) content in gWAT (*p < 0.001*), we next assessed mtDNA abundance in iWAT. In contrast to gWAT, males showed significantly higher mtDNA levels in iWAT compared to females (*p* = *0.024*; Figure 1B), indicating a depot-specific reversal in mitochondrial abundance. These results highlight that sex differences in mitochondrial content are not uniform across depots and suggest distinct regulatory mechanisms in iWAT vs. gWAT.

### 3.3 OXPHOS gene expression and mitochondrial respiration are female-biased in gWAT but male-biased in iWAT

To assess sex differences in mitochondrial gene expression, we analyzed transcripts encoding subunits of the oxidative phosphorylation (OXPHOS) complexes across fat depots. While gWAT exhibited widespread female-biased expression, iWAT showed moderate male-biased patterns (Figure 1C). To determine whether these transcriptional patterns were reflected at the functional level, we performed respirometry assays in gWAT and iWAT from SWR/J mice. As shown previously for other strains [21], female gWAT exhibited higher respiration for complex I (*log*_*2*_*FC = 1.2, p = 0.03*) and a trend toward higher complex IV activity (*log*_*2*_*FC = 2.0, p = 0.09*), while in iWAT, males showed a trend toward higher activity for both complex I (*log*_*2*_*FC = 0.85, p = 0.09*) and complex IV (*log*_*2*_*FC = 0.80, p = 0.19*; Figure 1D). These functional data align with the observed sex differences in mtDNA content and OXPHOS gene expression.

### 3.4 Mitochondrial DNA content in gWAT, but not iWAT is associated with metabolic traits in females

We then evaluated how mtDNA copy number correlates with metabolic traits across depots and sexes. In females, gWAT mtDNA levels inversely correlated with adiposity (*rho = -0.58, p < 0.001*), insulin, HOMA-IR (*rho = -0.39, p = 0.007*), and liver triglycerides (*rho = -0.34, p = 0.013*), consistent with a more favorable metabolic profile. In males, gWAT mtDNA also showed a significant inverse correlation with liver triglycerides (*rho = -0.45, p = 0.001*) and modest associations with adiposity (*rho = -0.31, p = 0.024*), and total cholesterol (*rho = -0.29, p = 0.044*). No significant associations were observed in iWAT for either sex. These findings, based on *n* = 3 per sex per depot, suggest a depot- and sex-specific relationship between mitochondrial abundance and systemic metabolism (Figure 1E).

### 3.5 Genetic regulation of mitochondrial genes is sex- and depot-specific

**Figure 2:**
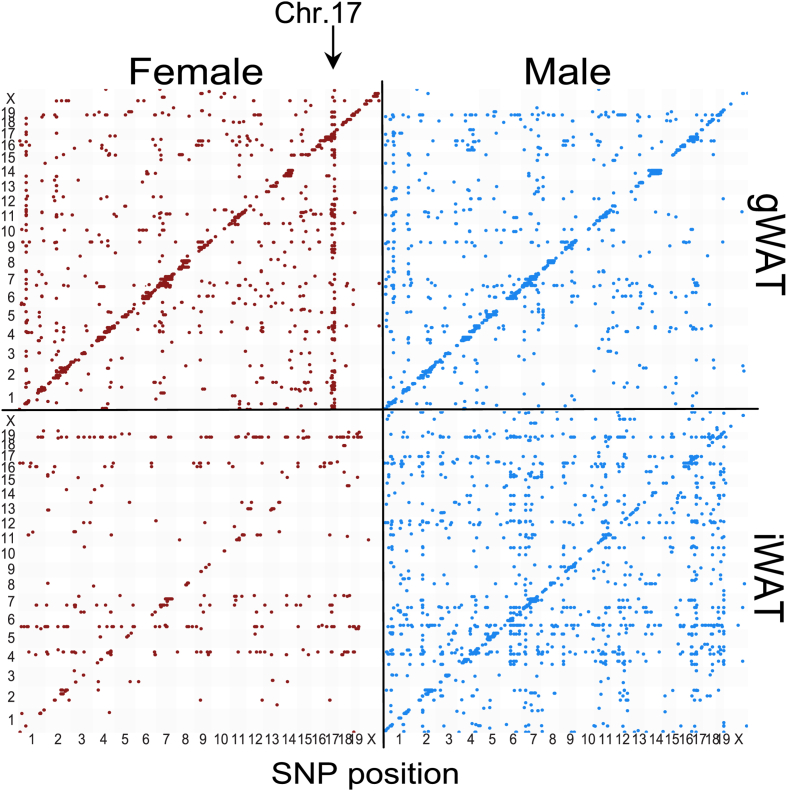
**Genetic mapping of mitochondrial gene expression reveals depot-specific *trans*-eQTL patterns.** Expression quantitative trait loci (eQTL) mapping of mitochondrial-related transcripts (MitoCarta3.0) was performed in gWAT and iWAT across HMDP strains (gWAT: 103 females, 112 males; iWAT: 67 females, 74 males). Each dot represents a significant SNP–gene association. *Cis*-eQTLs (*P* < 1 × 10^−5^), defined as SNPs within 1 Mb of the target gene, are shown along the diagonal. All other associations were considered trans-eQTLs (*P* < 1 × 10^−6^). Genetic mapping was performed using FaST-LMM.

To explore the genetic regulatory mechanisms, we examined expression quantitative trait loci (eQTLs). As previously reported [4], a strong female-specific *trans*-eQTL hotspot on chromosome 17, regulating *Ndufv2*, regulates 89 mitochondrial genes in gWAT, including many OXPHOS subunits, and is associated with higher mtDNA content and mitochondrial function in females. Notably, this *trans*-eQTL was not detected in iWAT of either sex, indicating that genetic regulation of mitochondrial pathways is both sex- and depot-specific (Figure 2).

### 3.6 Sex differences in futile cycle gene expression are depot-specific and cycle-specific

**Figure 3:**
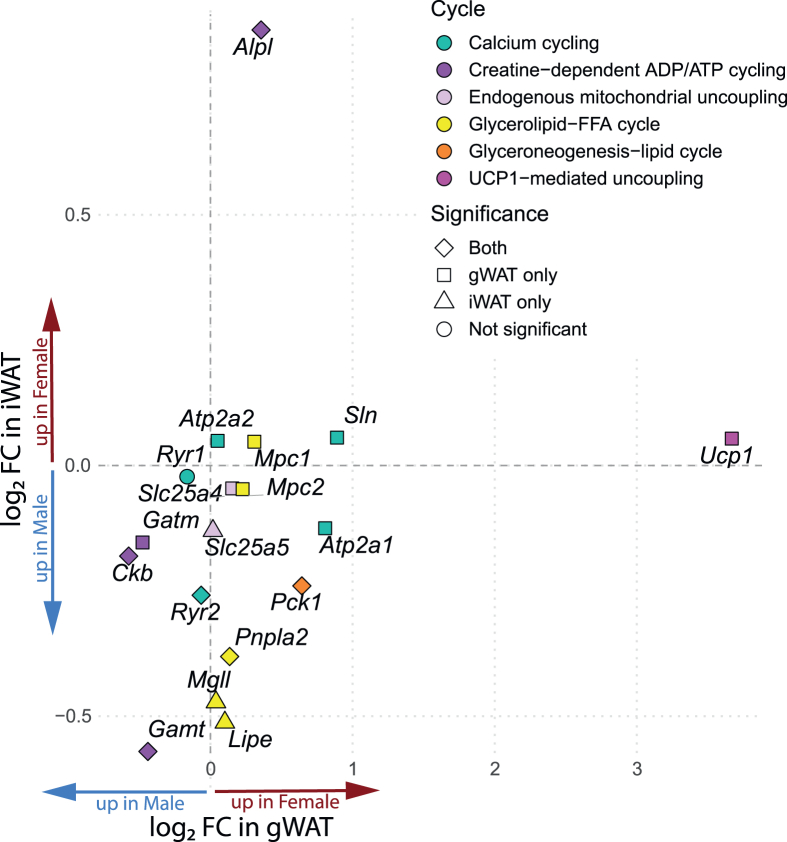
**Sex- and depot-specific differences in expression of ATP-consuming futile cycle genes.** Paired comparisons of gene expression between female and male mice in gWAT and iWAT for selected genes involved in ATP-consuming futile cycles. Values represent log_2_ fold change (female vs. male). Color indicates futile cycle category; symbol shape denotes significance based on paired t tests. iWAT, inguinal white adipose tissue; gWAT, gonadal white adipose tissue. (For interpretation of the references to colour in this figure legend, the reader is referred to the Web version of this article.)

Futile cycles, as described in [22], showed distinct, depot-specific patterns of activation in our sex-biased gene expression analysis (Figure 3). In gWAT, *Ucp1*, encoding the canonical mitochondrial uncoupling protein, showed the strongest female-enriched expression among all tested genes (*log*_*2*_*FC = 3.67*, *p = 1.2e-15*), alongside elevated expression of calcium cycling genes involved in SERCA- RyR- SLN- mediated thermogenesis, including *Sln*, *Atp2a1* (SERCA1), and *Atp2a2* (SERCA2b), indicating a robust female thermogenic signature in this depot. In contrast, iWAT showed no sex bias in *Ucp1* and only mild male-biased expression for calcium cycling gene Ryr2 (*log*_*2*_*FC = -0.26, p = 1.3e-4*), indicating depot-specific thermogenic regulation. A similar pattern appeared in human GTEx subcutaneous adipose tissue: modest female enrichment in *UCP1* expression (*log*_*2*_*FC* = *0.54, p = 0.03*), and strong male bias in *RYR2* (*log*_*2*_*FC* = *-1.86, p = 2.4e-6)*.

Interestingly, *Alpl*, encoding tissue-nonspecific alkaline phosphatase (TNAP)- recently identified as a key mitochondrial phosphocreatine phosphatase that drives the futile creatine cycle-was the most strongly female-biased gene in iWAT (*log*_*2*_*FC = 0.87*, *p = 2.6e-16*), and modestly enriched in female gWAT (*log*_*2*_*FC = 0.36*, *p = 7.8e-9*). This pattern suggests that TNAP-mediated creatine cycling may contribute to thermogenesis in female iWAT, potentially serving a complementary role in the absence of sex differences in *Ucp1* expression, despite its known female-biased expression in gWAT. Notably, other core genes of the creatine cycle (*Gamt*, *Gatm*, and *Ckb*), which regulate creatine synthesis and phosphorylation, were consistently male-biased in both depots. This highlights a potential functional divergence within the same pathway, where creatine synthesis enzymes are male-enriched, while the terminal ATP-consuming step via TNAP is female-enriched. Strikingly, this opposing female-enriched TNAP, male-enriched synthesis enzymes-was also observed in the GTEx database, supporting conserved sex dimorphism in creatine cycling (e.g., *log*_*2*_*FC*: *ALPL = 0.42, GATM/GAMT = -0.26, all p < 0.01*).

Beyond creatine cycling, we also examined other futile cycles relevant to lipid and energy metabolism. The glycerolipid-free fatty acid cycle also showed sex- and depot-specific regulation. In iWAT, male enriched expression of *Lipe* (encoding HSL), *Mgll* (MAGL), and *Pnpla2* (ATGL) suggests increased lipid cycling activity in males. Female gWAT showed higher expression of *Mpc1*, *Mpc2* (components of MPC1/MPC2 heterodimer responsible for transporting pyruvate into mitochondria), and *Pck1* (PEPCK-C, glyceroneogenesis–lipid cycle) suggests enhanced pyruvate import and glyceroneogenesis.

Together, these findings reveal depot- and sex-specific regulation of mitochondrial content and futile cycles, offering insight into the genetic and molecular mechanisms underlying adipose tissue function.

## 4 Discussion

This study extends previous findings on sex-specific regulation of adipose mitochondrial function by directly comparing gWAT and iWAT depots in a matched panel of genetically diverse mouse strains fed HF/HS diet. Despite comparable adiposity and fat distribution in 58 inbred mouse strains, male mice exhibited a more adverse metabolic profile, suggesting that sex differences in metabolic health are driven by intrinsic functional differences in adipose tissue rather than total fat mass or depot distribution. Notably, we observed a striking depot-specific inversion in sex-dependent mitochondrial and thermogenic gene regulation.

Earlier work established that females exhibit higher mitochondrial content and oxidative gene expression in gWAT [4]*,* a pattern that was not preserved in iWAT. Conversely, the mtDNA levels and OXPHOS gene expression were higher in male iWAT, indicating a depot-specific reversal in the direction of sex bias. Mitochondrial respiration followed a similar pattern: enhanced in female gWAT, as previously shown in other strains [21], and in male iWAT. These findings further emphasize that mitochondrial enhancement in gWAT is primarily linked to female metabolic protection. Notably, only gWAT mtDNA content correlated with improved metabolic traits in females, reinforcing the idea that mitochondrial function contributes to metabolic health in a depot- and sex-specific manner.

Previous work identified a strong female-specific *trans*-eQTL hotspot on chromosome 17 centered on *Ndufv2, that* regulates mitochondrial gene expression in gWAT, driving enhanced OXPHOS and mtDNA content in females [4]. This regulatory architecture was absent in iWAT. The lack of shared *trans*-eQTL architecture underscores the tissue-specific nature of genetic regulation, indicating that certain mitochondrial genes in iWAT are controlled by alternative pathways.

We next examined the expression of ATP-consuming futile cycle genes, implicated in thermogenesis and energy expenditure. iWAT is classically recognized as a beiging-prone depot with strong potential for browning and thermogenic activation in response to environmental or hormonal cues [14,15]. Under HF/HS conditions, however, we observed no sex difference in *Ucp1* expression, in contrast to the strong female-biased *Ucp1* expression in gWAT. This aligns with prior findings in chow-fed C57BL/6J mice, where females gWAT, but not iWAT, exhibited browning in response to β3-adrenergic stimulation, linked to greater sympathetic innervation [15]. Consistent with those findings, our data suggest that UCP1-mediated thermogenesis under metabolic stress remains largely restricted to female gWAT. Interestingly, *Alpl*, encoding TNAP, a key effector of the futile creatine cycle, was significantly higher in females in both depots, with a more pronounced sex difference in iWAT. Given that TNAP enables energy dissipation independent of UCP1 and is critical for thermogenesis in UCP1-deficient contexts [23,24], elevated *Alpl* in female iWAT may point to increased capacity for futile creatine cycle-mediated energy dissipation. In contrast, other core components of the creatine pathway (*Gamt*, *Gatm*, *Ckb*) were male-biased in both depots, suggesting that the synthetic machinery producing phosphocreatine (PCr) is more abundant in males, while the terminal, energy-dissipating step of the cycle (catalyzed by TNAP) is selectively enriched in females. While this suggests sex-specific decoupling of substrate supply and energy expenditure, functional validation (e.g., TNAP knockdown or thermogenic challenge studies) would be needed to confirm these mechanisms.

We observed significantly female-biased expression of *ALPL* in subcutaneous fat (*log*_*2*_*FC = 0.42, p = 2.7e-03*), alongside male-biased expression of the creatine biosynthesis enzymes *GAMT* and *GATM* (both *log*_*2*_*FC = -0.26; p = 1.4e-03* and *9.3e-03*, respectively) using age-adjusted, paired subcutaneous and visceral adipose tissue samples from the GTEx dataset. These results support a conserved regulatory mechanism across species and depots, though the functional consequences in humans remain to be explored. In male iWAT the expression of genes encoding key lipolytic enzymes involved in the glycerolipid/free fatty acid cycle was higher, suggesting enhanced capacity for lipid-fueled substrate cycling under nutrient-excess conditions. On the other hand, female gWAT displayed elevated expression of *Mpc1*, *Mpc2*, and *Pck1* suggesting enhanced pyruvate import and glyceroneogenesis. These data support a model in which female gWAT maintains tighter control of FFA flux via FFA recycling, while male iWAT may favor FFA release and substrate cycling as a means of energy dissipation. This pattern aligns with reports showing reduced MPC1 and MPC2 protein levels in iWAT from obese mice [25]. In humans, MPC1 expression was lower in subcutaneous adipose tissue from prediabetic females with impaired glucose regulation, while MPC2 was reduced in both sexes. Importantly, reduced MPC expression correlated with poorer glucose tolerance and higher adipose insulin resistance, highlighting a link between diminished MPC activity, impaired adipose function, and early features of metabolic disease [25]. The mitochondrial pyruvate carrier (MPC) facilitates pyruvate entry into mitochondria, where it serves as a key substrate for both de novo lipogenesis and glyceroneogenesis in WAT. These patterns support a model in which female gWAT maintains tighter control of FFA flux, while male iWAT is geared toward FFA release. Additionally, *Ucp1* and calcium cycling regulators (*Sln*, *Atp2a1*, *Atp2a2*) were elevated in female gWAT, supporting the concept that distinct combinations of futile cycles are deployed in a sex- and depot-specific manner to drive energy dissipation.

These differences may reflect underlying variations in hormonal signaling, sympathetic innervation, and mitochondrial function between fat depots. Thus, the coordinated activation of lipid, calcium, and creatine cycles across different fat depots and sexes highlights a flexible thermogenic system in which multiple pathways operate in parallel or compensate for one another depending on depot identity, sex, and physiological context. This supports the idea, that futile cycles are not ubiquitous but rather exhibit tissue- and sex-specific distributions, with their activity being context-dependent, inducible, and intimately regulated by environmental and hormonal cues.

This study has a few limitations. First, the use of bulk transcriptomic data does not allow resolution of cell type-specific gene expression within adipose tissue. Nonetheless, the observed sex- and depot-specific patterns were consistent and robust. Second, mitochondrial respiration was assessed in frozen samples from a single strain, which limits generalizability across the broader HMDP panel. However, the results aligned with transcriptomic differences, and in gWAT, they are further supported by data from fresh samples across multiple strains [21]. Third, the analysis focused on main sex effects without including interaction terms, in part due to limited power, although consistent patterns across depots support the findings. Finally, thermogenesis was not directly assessed *in vivo*, so the functional impact of the observed gene expression differences remains to be determined.

In summary, our systems genetics approach in the HMDP demonstrates that sex-specific regulation of adipose mitochondrial function and futile cycles is strongly depot-dependent. Genetically driven mitochondrial regulation in female gWAT, but not iWAT, appears central to female metabolic protection under HF/HS diet. Elevated *Alpl* expression in female iWAT may provide additional thermogenic flexibility through UCP1-independent mechanisms. Together, these findings suggest that female gWAT and male iWAT deploy distinct but complementary mechanisms to support metabolic flexibility and thermogenic potential, highlighting the importance of considering both depot and sex in adipose biology. While our findings highlight sex- and depot-specific regulation of mitochondrial function and futile cycles in mouse adipose tissue, and some of these patterns were also observed in human GTEx subcutaneous adipose tissue, extrapolation to humans should be done with caution. Mouse iWAT, the main subcutaneous depot, lacks a direct anatomical counterpart in humans, and the thermogenic capacity of human white adipose tissue is lower, with UCP1 expression largely limited in adults [1,2]. Future studies incorporating direct measurements of thermogenesis, gene-by-sex interaction models, and cell type-specific analyses may help uncover additional regulatory layers in iWAT and determine distinct mechanisms driving mitochondrial function in subcutaneous versus visceral fat.

## CRediT authorship contribution statement

**Dorota Kaminska:** Writing – review & editing, Writing – original draft, Visualization, Investigation, Formal analysis, Data curation, Conceptualization. **Calvin Pan:** Writing – review & editing, Formal analysis, Data curation. **Laurent Vergnes:** Writing – review & editing, Investigation, Formal analysis, Data curation. **Ashlyn Ro:** Writing – review & editing, Investigation. **Gurugowtham Ulaganathan:** Writing – review & editing, Investigation. **Aldons J. Lusis:** Writing – review & editing, Supervision, Resources, Project administration, Funding acquisition, Conceptualization.

## Authors' erelationships and activities

The authors declare that there are no relationships or activities that might bias, or be perceived to bias, their work.

## Contribution statement

DK conceived and designed the study, analyzed the data, and drafted the manuscript. CP contributed to data analysis and interpretation. LV performed and analyzed respirometry assays. AR and GU contributed to data collection. AJL supervised the project and contributed to manuscript revisions. All authors approved the final version.

## Funding statement

This work was supported by 10.13039/100000002NIH grants R01DK117850 and U54HL170326.

## Declaration of competing interest

The authors declare that they have no known competing financial interests or personal relationships that could have appeared to influence the work reported in this paper.

## Data Availability

Gonadal fat microarray data used in this study are available in the NCBI Gene Expression Omnibus (GEO) under accession number GSE64768. Raw RNA-sequencing data for inguinal adipose tissue are available in the NCBI Gene Expression Omnibus (GEO) under accession number GSE304104. Phenotypic data from the Hybrid Mouse Diversity Panel (HMDP) will be made available from the corresponding author upon reasonable request. GTEx datasets are publicly available from the GTEx Portal (https://gtexportal.org/home/datasets).
